# Eighteen-month-olds’ memory interference and distraction in a modified A-not-B task is not associated with their anticipatory looking in a false-belief task

**DOI:** 10.3389/fpsyg.2015.00857

**Published:** 2015-06-22

**Authors:** Norbert Zmyj, Wolfgang Prinz, Moritz M. Daum

**Affiliations:** ^1^Technical University of Dortmund, DortmundGermany; ^2^Max Planck Institute for Human Cognitive and Brain Sciences, LeipzigGermany; ^3^University of Zurich, ZurichSwitzerland

**Keywords:** false belief task, memory interference, infancy, distraction, inhibitory control

## Abstract

Infants’ performance in non-verbal false-belief tasks is often interpreted as if they have understood false beliefs. This view has been questioned by a recent account that explains infants’ performance in non-verbal false-belief tasks as the result of susceptibility to memory interference and distraction. We tested this alternative account by investigating the relationship between infants’ false-belief understanding, susceptibility to memory interference and distraction, and general cognitive development in 18-month-old infants (*N* = 22). False-belief understanding was tested in an anticipatory looking paradigm of a standard false-belief task. Susceptibility to memory interference and distraction was tested in a modified A-not-B task. Cognitive development was measured via the Mental Scale of the Bayley Scales of Infant Development. We did not find any relationship between infants’ performance in the false-belief task and the A-not-B task, even after controlling for cognitive development. This study shows that there is no ubiquitous relation between susceptibility to memory interference and distraction and performance in a false-belief task in infancy.

## Introduction

The proposal that infants are able to understand other agents’ false beliefs has been a source of lively debate over the last decade (Onishi and Baillargeon, 2005; Ruffman and Perner, 2005; Heyes, 2014; Scott and Baillargeon, 2014). In traditional false-belief tasks, children explicitly reason about an agent’s future behavior based on the agent’s false belief, which is indicated by the children’s verbal response (Wimmer and Perner, 1983). In the seminal “Maxi” task, Maxi puts the chocolate in the blue cupboard and leaves the room. While he is away, his mother enters the room and puts the chocolate in the green cupboard. Children are asked where Maxi will look for his chocolate after his return. Children demonstrate their false-belief understanding by indicating that Maxi will look for the chocolate in the blue cupboard. At around 4 years of age, children pass this type of task (Wellman et al., 2001).

The idea that children do not understand false beliefs before the age of 4 has been challenged by studies reporting that infants and toddlers are able to pass false-belief tasks if one uses tasks in which children react spontaneously and are not asked questions (see Onishi and Baillargeon, 2005, for the starting point of this debate). In an anticipatory looking task (Southgate et al., 2007), toddlers observed a hand puppet placing a ball in one of two boxes and an observing agent subsequently retrieving the ball in two familiarization trials. In order to reach into the box, the agent had to open one of two windows in a barrier. Each window was directly above the corresponding box. In a subsequent belief-induction trial, two different conditions were administered. The hand puppet placed the ball in the first box, which was observed by the agent in both conditions. Then, the hand puppet transferred the ball to the second box, which was observed by the agent in only one condition. Finally, the hand puppet took the ball out of the second box, which was not observed by the agent in both conditions. Accordingly, the agent held two different false beliefs regarding the location of the ball. Before the agent reached into one of the boxes, the toddlers’ eye gaze was measured. The majority of toddlers anticipated that the agent would reach for the ball in the location where she believed it to be. This type of task has also been employed with 18-month-olds (Thoermer et al., 2012). Although, here, the mean looking times revealed that infants did not look longer at the window that indicated false-belief understanding than at the other window, infants who did look longer at the correct window were more likely to pass standard false-belief tasks at 4 years of age. These findings of toddlers’ false-belief understanding are especially informative because action prediction is often seen as being more cognitively demanding than an evaluation of past behavior (Gredebäck and Melinder, 2010; Daum et al., 2012; Verschoor et al., 2013).

The mentalistic interpretation of infants’ performance in false-belief tasks has been controversial from the outset (e.g., Ruffman and Perner, 2005). However, the criticism only extended to single paradigms that reported evidence of infants’ false-belief understanding. Recently, this criticism has been articulated more comprehensively (Heyes, 2014). According to this view, young children’s performance in false-belief tasks is susceptible to low-level explanations for the following reasons: First, infants look longer at situations which they perceive to be novel (Olson and Sherman, 1983). For example, in Onishi and Baillargeon’s (2005) study, the agent reached into a green box, where she had not seen the object being transferred. Infants’ looking time was longer in this trial than in a trial in which the agent reached into a yellow box, where she had last seen the objects. Instead of attributing beliefs, infants might simply react to the novelty of the combination of person, place of the object, and reaching action. Second, infants’ memory might be affected by retroactive interference: If two events occur one after another, the memory of the latter event might interfere with the memory of the former. For example, an agent witnesses an object being placed into box A and then leaves the scene. The object is then transferred to box B before the agent reappears. The subsequent reappearance of the agent interferes retroactively with the memory of the transfer of the object into box B. Thus, infants might themselves believe that the object is in box A and expect the agent to look at box A. Third, disruptive elements of false-belief tasks might distract infants’ attention and therefore their memory.

The same argument might be applied to false-belief studies using anticipatory looking tasks. The ringing sound in Southgate et al.’s (2007) study, for example, might have distracted infants when the ball was being transferred from the first box to the second box (for the role distraction in imitation tasks, see Beisert et al., 2012). The agent’s head turn toward the boxes after the ball has been transferred might interfere retroactively with the memory of the transfer of the ball. Both processes would lead to the infants’ belief that the ball was still in the first box. Likewise, a deficit in working memory is fundamental to the A-not-B task because infants still look for a toy at location A even though they have observed the experimenter hiding the toy at location B. Although infants at the end of the first year of life master this task in the standard version, they fail if a delay is introduced between the hiding of the toy and the searching for the toy at location B (Diamond, 1985). Both proactive and retroactive interference might lead to this error. The repeated successful retrieval of the toy at location A might interfere proactively with the memory of the new location of the toy at B. Additionally, the delay between hiding the toy and searching for the toy might interfere retroactively with the memory that the toy is at location B. Thus, if a deficit in working memory is responsible for infants’ seemingly successful performance in a false-belief task, then we would expect that infants who fail in the A-not-B task in the B trials will succeed in the false-belief task.

In the present study, we therefore tested whether infants’ working memory is related to their performance in a false-belief task. We used an analogous version of Southgate et al.’s (2007) false-belief task and analyzed infants’ anticipatory looking via eye tracking. According to our knowledge, there are two infant studies suggesting that infants acknowledge the actors’ mental states in analogous videos. First, in Southgate et al.’s (2007) study, the majority of 25-month-olds anticipated that the actor directs her action to the box where she has last seen the object. Second, in Thoermer et al.’s (2012) study, 18-month-old performed at chance level in this task, but a correct anticipation at 18 months of age predicted passing the standard change-of-location task at 48 months of age. Another study with adult participants showed that adults with Asperger syndrome anticipated less reliably the reach of the actor according to her belief than adults without Asperger syndrome (Senju et al., 2010). We aimed at testing an age group with equal rates of passers and non-passers in the false-belief task. Southgate et al. (2007) reported that 85% of 25-month-old infants passed this test, while Thoermer et al. (2012) reported that only 55% of 18-month-olds passed. We opted to test 18-month-olds in order to increase the variance in infants’ performance, which is essential when comparing it to the performance in the A-not-B task.

A modified version of the A-not-B task was used in order to test infants’ working memory (Diamond, 1985) and retroactive interference (Heyes, 2014). After an object was hidden at one location and before the infants could reach for an object, a delay was introduced by putting a shield between the locations and the infants. This delay was reported to test infants working memory. For example, if landmarks indicate at which location a toy is hidden (location A or B), then infants do not err even after longer delays (Diamond, 1983). Additionally, lesions in the dorsolateral prefrontal cortex of macaques (Diamond and Goldman-Rakic, 1989) negatively affects their performance in the delayed A-not-B task and this region is typically associated with higher executive functions such as working memory (Stern et al., 2001). The delay is also believed to introduce retroactive interference: Putting the shield between the infant and the hidden object weakens the memory for the event that happened before, namely, the object being hidden at one location (Heyes, 2014). We further controlled for infants’ cognitive development by employing the Mental Scale of the Bayley Scales of Infant Development (BSID-II).

## Materials and Methods

### Participants

Participants were 22 eighteen-month-olds (*M* = 18 months; 2 days, SD = 0;08; age range: 17;14–18;15, 14 girls). Thirty-nine additional infants were tested but excluded from the final sample due to fussiness and lack of interest during the false-belief task (*n* = 15), the A-not-B task (*n* = 9), or the Mental Scale of the BSID-II (*n* = 2). Further reasons were procedural errors (*n* = 6), failing to meet the inclusion criterion in the false-belief task (*n* = 4), interference by the parent (*n* = 1), or equipment failure (*n* = 1). Although the attrition rate is high, it is analogous to similar studies on false-belief understanding in this age range (e.g., Southgate et al., 2007; Buttelmann et al., 2009). The experiment was conducted in accordance with the ethical standards laid down in the Declaration of Helsinki and the standards of the local ethics committee of the University of Leipzig.

### Material

The false-belief task was presented and gaze was measured using a Tobii 1750 near infrared eye tracker with an infant add-on (precision: 1°, accuracy: 0.5°, sampling rate: 50 Hz). A 9-point infant calibration was used. Viewing distance was approximately 80 cm. In the A-not-B task, a wooden panel (40 cm × 10 cm) and two plastic cups were used. An upright board (height = 25 cm, width = 45 cm) obscured infants’ view of the cups. A maximum of sixteen cubes (3 cm × 3 cm × 3 cm) could be retrieved by the infants and put into a xylophone box, resulting in a series of tones.

### Design

All infants were tested in three tasks: the false-belief task, the A-not-B task, and the BSID-II. The order of the false-belief task and the A-not-B task was counterbalanced and the BSID-II was conducted last.

### Procedure

#### False-Belief Task

In the false-belief task, infants viewed videos, which presented two familiarization trials and one test trial. In all videos, an actor sat behind a board that contained a left and a right window. He wore a white visor cap and moved his head as if he were following the displayed actions closely in order to increase the impression that he was being attentive. Infants also watched a second similar video, which was presented before or after the false-belief task. However, this video was beyond the scope of the present research question and is not reported here.

**FIGURE 1:**
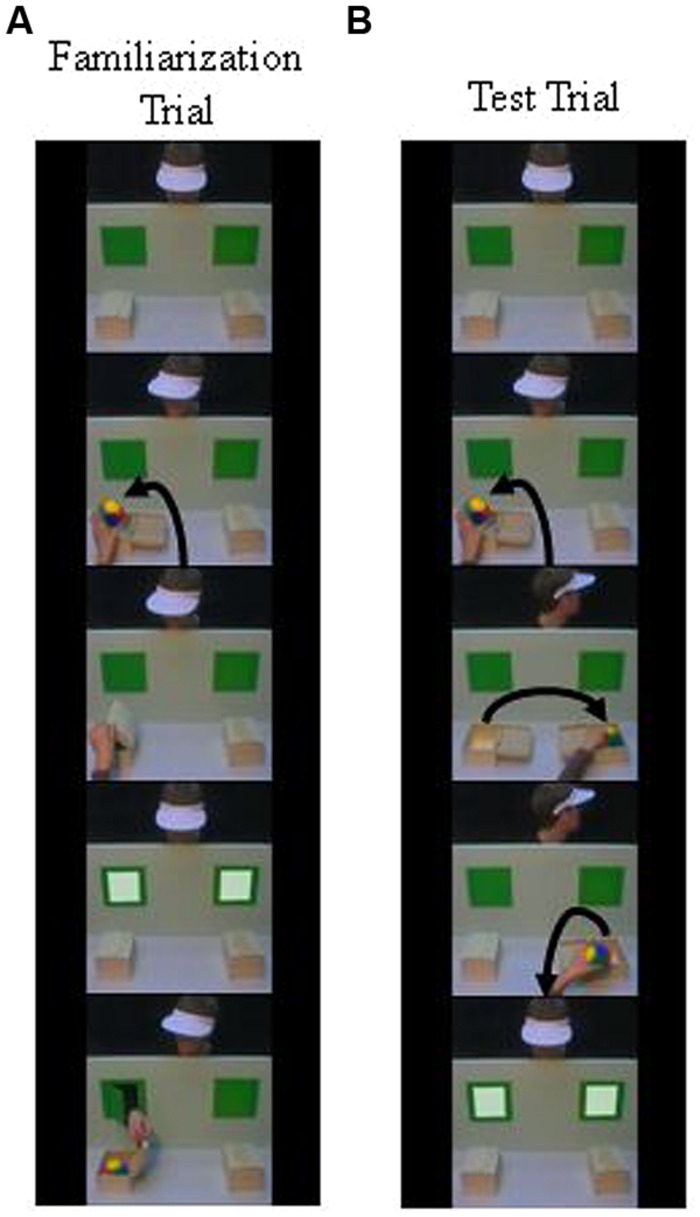
**Successive frames from the videos of one of the familiarization trials **(A)** and the test trial **(B)** for the change-of-location task**.

The task was analogous to Southgate et al.’s (2007) false-belief II task: An opaque box was placed in front of each of the two windows described above. In the familiarization trials (see **Figure 1A**), the actor witnessed a ball being hidden by a human hand (henceforth called “operating hand”) in one of the two boxes. The duration of this sequence was 10 s. Then, in an anticipation phase, which was similar across trials and tasks, a chime sounded and a still image was presented, with both windows first being illuminated (1,000 ms) and then not illuminated (1,750 ms). During the anticipation phase it was measured whether infants fixated the two windows. Each area of interest was 7.55 cm × 5.44 cm (height × width) which equals a visual angle of 5.4° × 3.9°. The actor then reached through the window on the side where the ball was located and opened the box (duration = 6 s). In the test trial (**Figure 1B**), the ball was again hidden in one box (duration = 10 s). Then, a telephone started ringing and the actor turned around. While he was looking away, the operating hand transferred the ball from one box to the other and after that, removed the ball from the second box (duration = 30 s). Then, the telephone stopped ringing, and the actor turned back and the next anticipation phase started.

We only analyzed infants’ looking behavior in the test trial if infants met the inclusion criterion applied by Thoermer et al. (2012). Infants’ looking time to the correct window had to be longer than infants’ looking time to the incorrect window in at least one familiarization trial. It should be noted that the same pattern of result showed when we used the inclusion criterion (i.e., toddlers had to direct their first fixation in the second familiarization trial to the correct window) applied by Southgate et al. (2007).

#### A-not-B Task and BSID

A modified version of the A-not-B task was designed for 18-month-olds (Diamond, 1985). Infants sat on their parents’ lap facing the experimenter who sat at the opposite side of table. The experimenter hid a small cube under one of two cups. He then hid the cups behind the occluder for 5 s and finally placed them within the infants’ reach. If the infants successfully retrieved the cube, they could put it in the xylophone box. After four successful retrievals from location A, the hiding place was switched to location B. The experiment ended when infants found the cube at location B. Finally, infants’ cognitive development was assessed using the Mental Scale of the BSID (2nd edition, Bayley, 1993).

### Coding and Reliability

In the false-belief task, infants’ looking behavior was analyzed for 2,750 ms after the anticipation phase began. The first fixation on one of the two windows lasting more than 200 ms was identified and categorized as (a) anticipatory fixation that was congruent with the actor’s false belief, resulting in a score of 1, or (b) anticipatory fixation that was incongruent with the actor’s false belief, resulting in a score of 0. Additionally, the duration of all fixations on both windows during the anticipation phase was assessed and transformed into two sum scores (i.e., one sum score for each window). The proportion of mean looking time at one window was calculated by dividing the looking time at one window by the sum of looking times at both windows.

In the A-not-B task, the coding began when infants had retrieved the cube in four successive trials from location A and the cube was hidden at location B. We counted the number of trials in which infants searched at location A before they finally searched at location B.

The reliability rating of the A-not-B task by a second independent rater was excellent (*r* = 0.92, intraclass correlation coefficient). Infants’ performance in the BSID was analyzed according to the standard procedure as described in the BSID manual.

## Results

### False-Belief Task

Infants’ first fixation on the location that corresponded to the actor’s false belief (7 out of 22 infants, *M* = 32%) did not differ from the chance level (*p* = 0.13, binomial test)^1^. Analyses of the mean looking time of all fixations on the first box (where the agent has seen the ball being transferred) and the window above and as well as the second box (where the agent has not seen the ball being transferred) and the window above during the anticipation phase of the test trial did not result in any statistically significant effect [*M*_firstbox_ = 533 ms, SD = 479 ms, *M*_second box_ = 913 ms, SD = 682 ms, *t*(21) = 1.77, *p* = 0.09]. The infants’ first anticipatory look to the side where the actor last saw the ball and proportion of looking the side where the actor last saw the ball correlated (*r* = 0.77, *p* < 0.001). Further analyses of possible moderating variables such as mean looking time toward the videos before the anticipation phase, referential looks toward the actor’s head, and number of correct anticipatory looks during the familiarization trials did not reveal any statistically significant findings.

### A-not-B Task and BSID

The mean number of incorrect searches at location A was 1.4 (SD = 1.7, range 0–5). Nine infants did not perform an incorrect search at location A, six infants searched at location A one time, two infants each searched at location A two, four, and five times, and one infants searched at location A three times. Infants’ mean IQ score in the BSID was 93.3 (SD = 10.3, range 79–117). Four infants received a score below 85, 17 infants received a score within 85 and 115, and one infant received a score above 115. In order to assess whether infants who were excluded performed worse in the A-not-B task than infants who were included in the study we compared their performance. We did not find a difference between both mean scores, *M*(excluded) = 1.4 (SD = 1.6), *t* < 1.

### Relationship between Tasks

**FIGURE 2:**
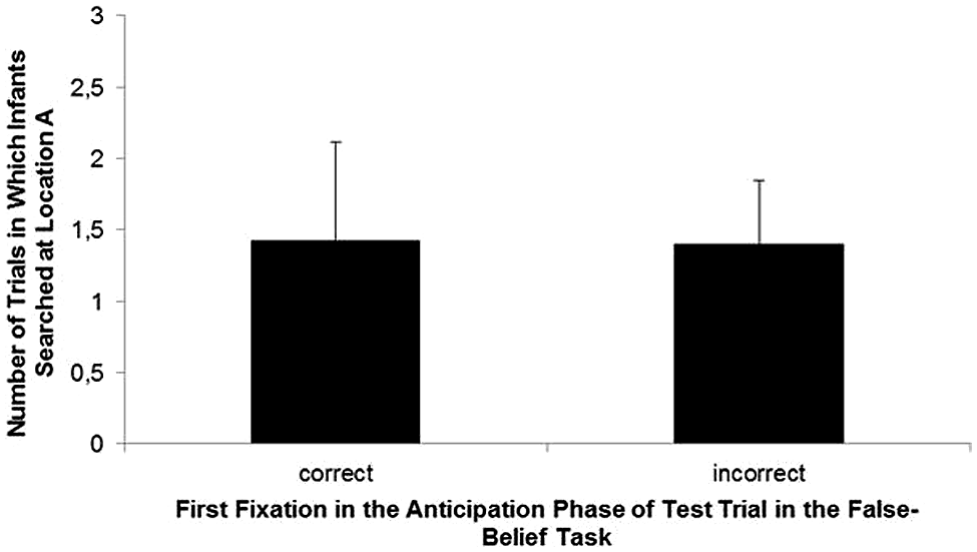
**Number of incorrect searches at location A in the A-not-B task for infants whose first fixation in the false-belief task was directed to the location where the actor last saw the ball (correct) and whose first fixation was directed to the location where the actor did not last see the ball (incorrect)**.

There was no statistically significant relationship between the false-belief task and the A-not-B task for the toddlers’ first fixation in the false-belief task (*r* = -0.02, *p* = 0.92, Spearman rank correlation, see **Figure 2** for group differences between infants who anticipated correctly and incorrectly in the false-belief task) or for the proportion of mean looking time of fixations on the first box – where the agent has seen the ball being placed (*r* = 0.11, *p* = 0.63, Spearman rank correlation). This remained after controlling for cognitive development measured in the BSID (*r* = -0.06, *p* = 0.78; *r* = -0.15, *p* = 0.49, Spearman rank correlation).

## Discussion

The present study revealed no correlation between a non-verbal false-belief task and a modified version of the A-not-B task. The lack of correlation remained after controlling for infants’ cognitive development. Accordingly, the present study provided no evidence for the assumption that a deficit in working memory is related to infants’ success in a non-verbal false-belief task (Heyes, 2014). This null result is especially informative because the scenario in the false-belief tasks was suggested to resemble the A-not-B task (Heyes, 2014) and a considerable proportion of infants failed in both tasks, which opened up the possibility that performance in both tasks correlated.

The absence of evidence of a relationship between performance in a working memory task and a false-belief task is not evidence of the absence of this relationship. Accordingly, this study does not straightforwardly refute the proposal that low-level explanations apply to false-belief tasks for infants. There are at least two possible explanations for the lack of relationship between the two tasks in the present study. A first explanation is based on specific characteristics of the tasks in this study, which might have reduced the correlation between the false-belief task and the A-not-B task. The critical variables might have been the infants’ age, the criterion for anticipatory looking, and the stimuli used in the false-belief task. Whereas in the original study, 25-month-olds were tested (Southgate et al., 2007), we chose 18-month-olds in order to obtain more variance in their anticipatory looking. Previous findings have shown that 18-month-olds’ looking behavior is not random in this task, but that the false-belief-like looking pattern (i.e., gaze to the window according to the agent’s false belief) is related to their explicit false-belief understanding at the age of 4 years (Thoermer et al., 2012). Next, the criterion for an anticipatory fixation on one of the two windows was that it lasted more than 200 ms. We used this criterion because fixations with shorter duration appeared to be random and not directed to the window. However, when applying the original criterion by Southgate et al. (2007) of more than 20 ms, the correlation between the false-belief task and the A-not-B task remained statistically not significant. Additionally, although we matched the stimuli closely to the original stimuli used in Southgate et al.’s (2007) study, there were minor differences. The agent in the present study was male, while the agent of the Southgate et al. (2007) study was female. In the present study, a human hand transferred the ball from one location to the other, whereas in Southgate et al.’s (2007) study, a hand puppet resembling a polar bear transferred the ball. However, we do not think that these differences affected the infants’ working memory, and therefore do not believe them to be responsible for the lack of correlation between performance in the false-belief task and the A-not-B task. Finally, we tested infants’ false-belief understanding in an anticipatory-looking task which represents only one measure to test infants’ false-belief understanding. We did not test infants’ false-belief understanding in a violation-of-expectation task. There is a structural difference between both tasks because in anticipatory-looking tasks infants have to predict an agent’s action whereas in violation-of-expectation tasks, infants have to evaluate an executed action. It remains an open question whether working memory and distraction is the key to understand infants’ performance in false-belief tasks in violation-of-expectation tasks.

A second explanation is based on a mentalistic interpretation of infants’ performance in the false-belief task. If infants do attribute false beliefs to others, then it is not surprising that this ability is not closely related to their working memory. Working memory might be a prerequisite for remembering the story line of a false-belief scenario. The mere memory, however, does not imply insight into the mental states of others. This notion was supported by a meta-analysis showing that early executive functions predict later false-belief understanding but not vice versa (Devine and Hughes, 2014).

The idea that young children are able to infer others’ mental states is thought-provoking and should be tested more rigorously in the future. Improving the false-belief scenarios is one strategy to test this question (Heyes, 2014). In the present study, we used another strategy by correlating an established false-belief task with the A-not-B task, which tests inhibitory control and working memory. This strategy has been applied in previous studies on the relationship between non-verbal belief tasks and other tasks on inhibitory control, which were identified as being closely related in standard verbal false-belief tasks (for an overview, see Devine and Hughes, 2014). These studies revealed mixed findings. Three- and 4-year-olds’ performance in the Dimensional Card Change Sorting task and a non-verbal false-belief task showed no relationship (Low, 2010). In contrast, 18-month-olds’ performance in a detour task (i.e., infants had to open a box with a transparent window presenting a toy by touching a knob attached to the side of the box) and a non-verbal false-belief task using a violation-of-expectation paradigm did show a relationship (Yott and Poulin-Dubois, 2012). The latter finding is surprising because non-verbal false-belief tasks were designed to eliminate inhibitory control demands (Baillargeon et al., 2010). However, we were unable to find this relationship between the A-not-B task and a false-belief task in an unexpected transfer scenario. The present findings might indicate that predictive eye gaze is less affected by infants’ inhibitory control than looking times in violation-of-expectation paradigms. A possible reason could be that predictive eye gaze is a more automatic response than continuous looking to an event, and therefore predictive eye gaze is less targeted by higher cognitive processes such as inhibition. It is clear, however, that the relationship between false-belief understanding in infancy and executive functions including working memory and inhibitory control should be further investigated.

The present study replicated Thoermer et al.’s (2012) finding that 18-month-olds as a group do not perform above chance level when anticipating an agent’s action based on the agent’s false belief. Southgate et al. (2007) demonstrated that the majority of 25-month-olds pass this test which shows that between 18 and 25 months of age infants develop the ability to anticipate others’ action based on the others’ false beliefs. The fact that children who passed the false-belief test at 18 months of age were more likely to pass a standard false-belief test at 48 months of age indicates that infants at 18 months of age do not perform at random, but that some 18-month-olds are sensitive to another’s false belief. In the present study, we showed this successful performance in 18-month-olds is not predicted by distraction or memory interference as measured by the modified A-not-B task.

In sum, the present study revealed no relationship between performance in a false-belief task using an anticipatory looking paradigm and a modified version of the A-not-B task even after controlling for cognitive development. Accordingly, this study finds no evidence in support of a relationship between working memory and false-belief understanding in infancy.

## Conflict of Interest Statement

The authors declare that the research was conducted in the absence of any commercial or financial relationships that could be construed as a potential conflict of interest.

## Footnotes

1 A similar result was obtained when applying the original coding criterion of Southgate et al. (2007), who coded an anticipatory look to one location if the infant fixated on the AOI for more than 20 ms.
